# Development and validation of epigenetic modification-related signals for the diagnosis and prognosis of colorectal cancer

**DOI:** 10.1186/s12864-023-09815-2

**Published:** 2024-01-11

**Authors:** Xia Li, Jingjing Li, Jie Li, Nannan Liu, Liwei Zhuang

**Affiliations:** 1 Department of Gastroenterology and Hepatology, The Fourth Affiliated Hospital of Harbin Medical University, Harbin, 150001 Heilongjiang Province China; 2 Department of Endocrinology and Metabolism, The Second Affiliated Hospital of Harbin Medical University, Harbin, 150086 Heilongjiang Province China; 3 Department of Neurology, The Second Affiliated Hospital of Harbin Medical University, Harbin, 150086 Heilongjiang Province China

**Keywords:** Colorectal cancer, Epigenetic-related genes, Bioinformatics, Risk score, Prognosis

## Abstract

**Background:**

Colorectal cancer (CRC) is one of the world's most common malignancies. Epigenetics is the study of heritable changes in characteristics beyond the DNA sequence. Epigenetic information is essential for maintaining specific expression patterns of genes and the normal development of individuals, and disorders of epigenetic modifications may alter the expression of oncogenes and tumor suppressor genes and affect the development of cancer. This study elucidates the relationship between epigenetics and the prognosis of CRC patients by developing a predictive model to explore the potential value of epigenetics in the treatment of CRC.

**Methods:**

Gene expression data of CRC patients’ tumor tissue and controls were downloaded from GEO database. Combined with the 720 epigenetic-related genes (ERGs) downloaded from EpiFactors database, prognosis-related epigenetic genes were selected by univariate cox and LASSO analyses. The Kaplan–Meier and ROC curve were used to analyze the accuracy of the model. Data of 238 CRC samples with survival data downloaded from the GSE17538 were used for validation. Finally, the risk model is combined with the clinical characteristics of CRC patients to perform univariate and multivariate cox regression analysis to obtain independent risk factors and draw nomogram. Then we evaluated the accuracy of its prediction by calibration curves.

**Results:**

A total of 2906 differentially expressed genes (DEGs) were identified between CRC and control samples. After overlapping DEGs with 720 ERGs, 56 epigenetic-related DEGs (DEERGs) were identified. Combining univariate and LASSO regression analysis, the 8 epigenetic-related genes-based risk score model of CRC was established. The ROC curves and survival difference of high and low risk groups revealed the good performance of the risk score model based on prognostic biomarkers in both training and validation sets. A nomogram with good performance to predict the survival of CRC patients were established based on age, NM stage and risk score. The calibration curves showed that the prognostic model had good predictive performance.

**Conclusion:**

In this study, an epigenetically relevant 8-gene signature was constructed that can effectively predict the prognosis of CRC patients and provide potential directions for targeted therapies for CRC.

**Supplementary Information:**

The online version contains supplementary material available at 10.1186/s12864-023-09815-2.

## Introduction

Colorectal cancer (CRC) is one of the top three causes of tumor-related deaths as shown in global cancer statistics [1]. Colorectal cancer can be treated with surgery, chemotherapy, radiotherapy, and other biological immunological therapies [2]*.* Surgery is the first line of treatment, but CRC patients are risk of poor prognosis [3]. Colorectal cancer‘s pathogenesis remains unknown due to variety of pathogenic factors, which makes treatment more difficult [4]. Thus, further research to investigate the underlying mechanisms of CRC onset and progression is essential for subsequent therapeutic studies. Researchers have discovered more mechanisms leading to tumorigenesis in recent years, with epigenetic modifications playing a part in cancer development and progression [5]. Studies have shown that epigenetic modifications, including aberrant DNA methylation, are important during CRC development [6]. Therefore, a number of epigenetic biomarkers may help predict and diagnose CRC, as well as provide prognosis [7]*.*

An epigenetic change is a separate change of DNA sequences, which is heritable and dynamic at the same time [8]. There is growing evidence that epigenetic modifications are important in the treatment of cancer [9, 10], and it is thought to play an important function in carcinogenesis and cancer progression [11]. Now aberrant epigenetic modifications affect cancer initiation and progression. Epigenetic changes have also been identified to play a key function in the development and progression of colorectal cancer [12–15]. Recent data have reported that epigenetic changes are closely related to tumor transformation in CRC [16, 17]. In recent years, abnormal DNA methylation has become the most studied epigenetic modification due to its close connection with tumorigenesis and progression through repair of tumor suppressor genes [18]. As a result, epigenetic modifications can affect many phenotypic characteristics in tumor cells, including growth, immune escape, metastasis, heterogeneity, and chemoresistance [19]. In addition, a sufficient amount of research has been done on the part of histone methylation in the development of digestive cancers [20]. The study of histone modifications in colorectal tumorigenesis has provided new insights for therapeutic targets [21]. Karczmarski et al. study demonstrated that significantly increased level acetylation of H3K27 in CRC samples compared with normal tissue [22]. Most colorectal tumors are adenocarcinomas originating from benign adenomatous polyps. Research suggests that epigenetic changes are associated with aberrant crypt foci (ACF)-adenomas-carcinomas, which is vital to the CRC development [23]. Vogelstein et al. [24] has proved that a genetic adenoma-tocarcinoma sequence model for colon tumorigenesis in 1988. Epigenetic alterations have now been associated with specific links in the adenoma-carcinoma sequence, and are thought to play an essential part in the pathogenesis of CRC [25, 26]. However, it would have been better if the studies have focused on the functional extensive exploration.But, it is unclear whether these genes have any value in diagnosing and prognosing CRC. In the study, it has been found that an epigenetic-related eight-gene signature is capable of predicting prognosis and survival time in CRC patients.

## Materials and methods

### Data source

The mRNA sequencing data of 203 CRC and 160 control samples in the GSE87211 dataset was downloaded from Gene Expression Omnibus (GEO) database (https://www.ncbi.nlm.nih.gov/geo/), and was used to screen differentially expressed genes (DEGs). The GSE40967 dataset containing the RNA sequencing data and clinical survival information of 585 CRC patients was used for prognostic analysis and construction of the prognostic model. The GSE17538 dataset served as a validation set with gene expression profiles and survival information for 238 CRC patients. 720 epigenetic-related genes (ERGs) were obtained from EpiFactors database (http://epifactors.autosome.ru) [27].

### Acquisition of epigenetic-related DEGs in CRC and functional enrichment analysis

The DEGs between normal and tumor groups in the GSE87211 dataset were analyzed and visualized by the “DESeq2” package [28] with adj.P.Val < 0.05 and |Log2FC|> 1. We overlapped DEGs and ERGs to obtain epigenetic-related DEGs (DEERGs). To reveal the functions of DEERGs, R “clusterProfiler” package was used for Gene Ontology (GO) annotation [29] and Kyoto Encyclopedia of Genes and Genomes (KEGG) enrichment [30] analyses. The location of DEERGs on chromosomes was analyzed and displayed using the R “OmicCircos” package.

### Establishment and validation of the prognostic model

We used gene expression data and clinical information from GSE40967 to construct the risk model. Univariate Cox regression was used to analyze the DEERGs obtained in the previous step, and set a threshold *P* < 0.05 to screen for prognosis-related genes in CRC. Afterwards, LASSO regression analysis was performed using “glmnet” package to further obtain prognosis module genes. Based on the expression of prognosis module genes and the risk coefficient (coef) obtained, CRC cohorts were categorized as two risk groups (high and low) via the median risk score. Kaplan–Meier (KM) survival curves and receiver operating characteristic (ROC) curves were plotted to assess the prognostic value of risk characteristics using the R packages “survivor” and “survivorROC”, respectively. The risk model was validated in the GSE17538 dataset.

Thereafter, clinicopathological features and risk scores were incorporated into univariate and multivariate cox regression analysis to screen independent prognostic factors, and a nomogram of them was plotted via the “rms” package to predict the survival probability of CRC patients in the TCGA dataset at 1-, 2- and 3 years. Otherwise, the corresponding calibration curve was also drawn to assess the validity and dependability of the nomogram.

### Gene set variation analysis (GSVA)

To further explore the potential biological functions of genes in different risk groups (high and low), the “GSVA” package was used to perform GSVA pathway analysis. The adj.p.val < 0.05 was used to screen for significantly enriched pathways. 

### Evaluation of the immune microenvironment landscape

The ESTIMATE algorithm provided in the R package “ESTIMATE” was used to calculate the immune and stromal scores of CRC samples to predict the immune and stromal components of the tumor [31]. In addition, a correlation analysis of risk scores with immune and stromal scores was implemented by Spearman correlation analysis. Then CIBERSORT database was used to evaluate the immune infiltration level of patients and screen the differential immune cells between low- and high- risk groups. Moreover, differential analysis was also performed on the expression levels of immune checkpoints genes in different risk groups by Wilcoxon test.

### Correlations of risk model genes with m6A and m5C associated genes

The differential m6A modifiers and m5C regulators between high- and low-risk groups were recognozed via Wilcoxon test. 19 m6A modifiers included “writers” WTAP, METTL14, ZC3H13, RBM15, CBLL1, METTL3, “erasers” ALKBH5. I, FTO and “readers” RBMX, YTHDF1, FMR1, YTHDC2, YTHDC1, IGF2BP1, YTHDF3, IGF2BP2, YTHDF2, ELAVL1, HNRNPA2B1, TRA2A. Moreover, 20 m5C regulators included “readers” ZBTB33, MBD1, MBD4, NTHL1, SMUG1, TDG, UHRF1, UHRF2, MECP2, UNG, NEIL1, ZBTB38, MBD3, ZBTB4, and MBD2, “writers” DNMT3A, DNMT1, and DNMT3B, and “erasers” TET3, TET1, and TET2. Subsequently, the relevance of risk model genes to m6A modifiers and m5C regulators was analyzed by Spearman correlation analysis. The “ggplot2” package was utilized to visualize the results.

### Drug prediction

To mine the potential drug target information for module genes, we uploaded them into the DGIdb database (www.dgidb.org) to access potential therapeutic drugs for CRC patients [32].

### Quantitative Real-Time Polymerase Chain Reaction (qRT-PCR)

Endoscopy of CRC patients at the Fourth Affiliated Hospital of Harbin Medical University was used to obtain human CRC samples. TRIzol reagent was used to extract total RNA from human CRC (Beijing Solarbio Science & Technology Co., Ltd.). The mRNA expression levels of NAP1L2, HDAC9, SATB2, TONSL and CHAF1B in the20 pairs of human CRC and adjacent tissues were detected by RT-PCR. The primer sequences for qRT-PCR were as follows: NAP1L2 primers 5-GTTCTCAAAGCCTCAGCACCA-3 and 5-CAAAGGACCGTACACGCCTAA -3; HDAC9 primers 5-CTTGTAGCTGGTGGAGTTCCC-3 and 5-CTCTGTCTTCTTGCATCGCCT-3; SATB2 primers 5-GGAGGAGTCAAGGCATCACC -3 and 5- GCCTTCCTCGCTGTCGTTCT-3. TONSL primers 5-GCAGAGCAATGACGAGGTGTT -3 and 5- TGCGGTAGCGGTCAGTCAA-3. CHAF1B primers 5-GATGAGTCTGCCCTACCGC -3 and 5- AACTTGGTGGAGTGTCCGTCTT-3. The cycle threshold (Ct, which is the inflection point on the amplification power curve) was calculated, and the 2 − ΔΔCT method was used to calculated relative gene expression [33]. The Actin was used as the internal reference gene, and the primer sequences were listed in Supplementary Table 1.

## Results

### Identification of DEERGs and functional enrichment analysis

**Fig. 1:**
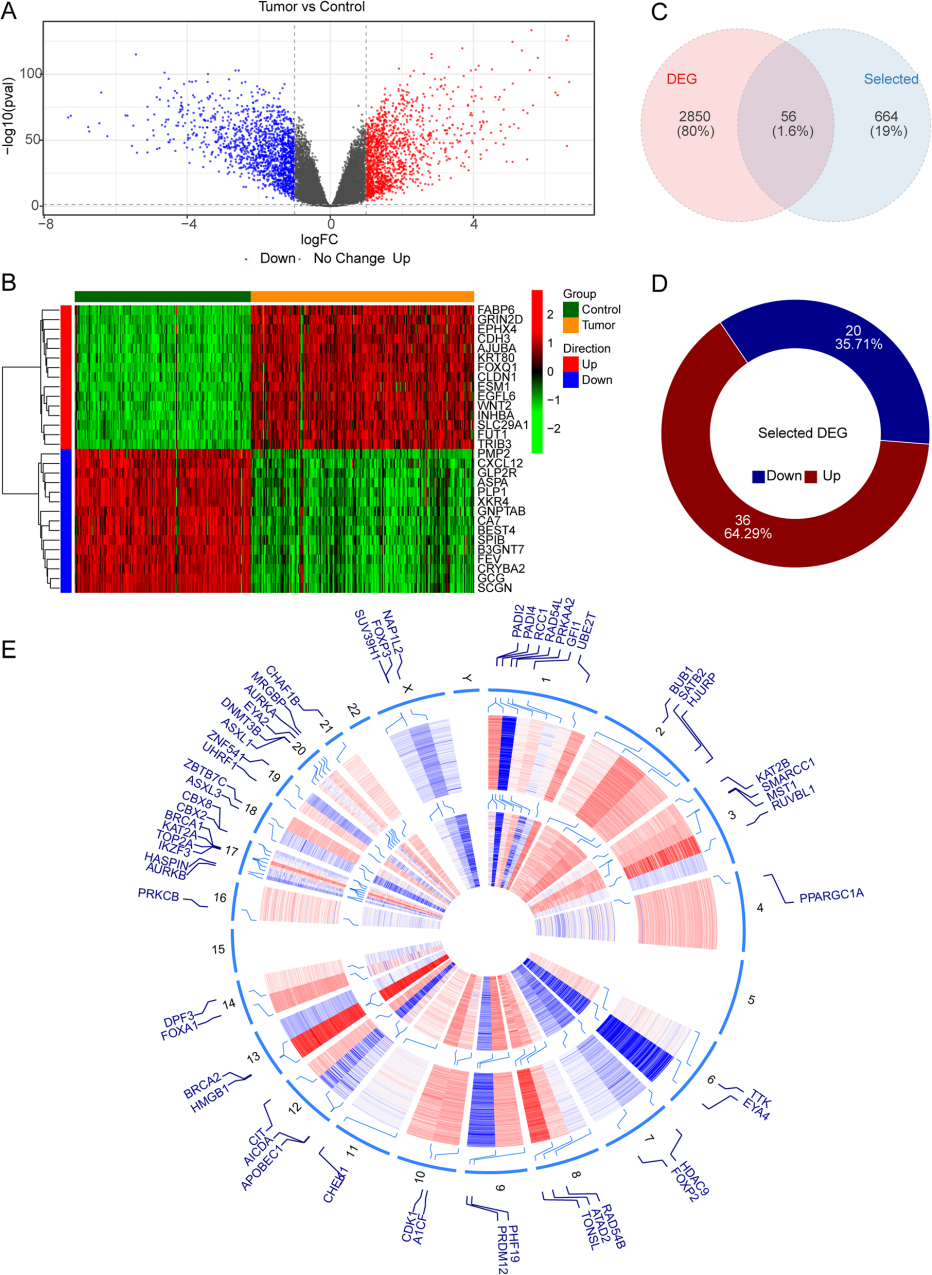
Systematic analysis of epigenetic-related genes. **A** Volcano maps for 2906 differentially expressed genes (DEGs) based on Gene Expression Omnibus (GEO) database. **B** Heatmap of DEGs between colorectal cancer (CRC) and normal tissues. **C** A total of 2906 DEGs were identified from GSE87211 dataset. After overlapping DEGs with 720 epigenetic-related genes (ERGs) and we obtained 56 differentially expressed ERGs (DEERGs). **D** 56 DEERGs were identified from GSE87211, including 36 upregulated genes and 20 downregulated genes. **E** The locations of the 56 DEERGs on chromosomes

By comparing tumor and normal tissue samples, there were 2906 genes differentially expressed, where 1384 DEGs up-regulated and 1522 DEGs down-regulated (Fig. 1A). The heat map shows the expression of the first 15 up-regulated and down-regulated genes (Fig. 1B). After overlapping DEGs with 720 ERGs, we obtained 56 DEERGs (Fig. 1C). In tumor samples, 36 of 56 DEERGs were up-regulated and 20 were down-regulated (Fig. 1D). The locations of the 56 DEERGs on chromosomes were shown in (Fig. 1E).

**Fig. 2:**
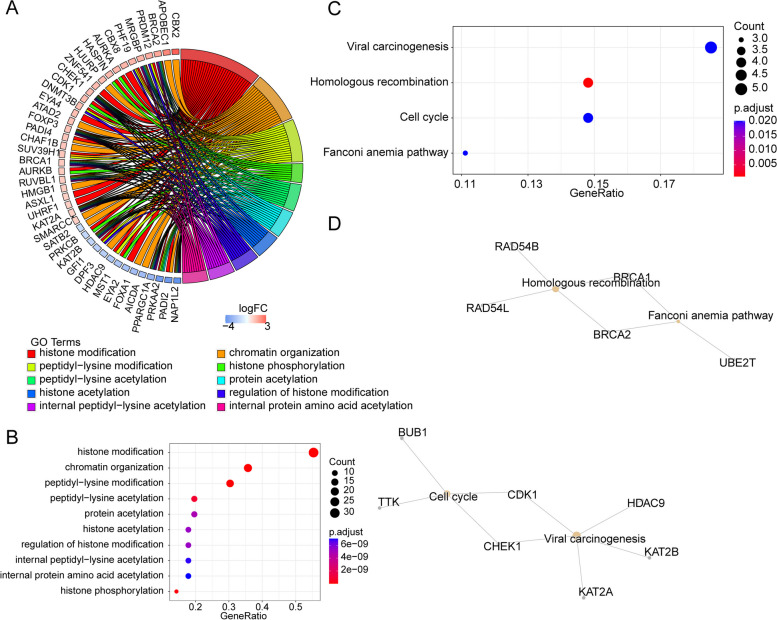
Functional enrichment analysis of DEERGs. **A** Gene Ontology (GO) analysis of 56 DEERGs. **B** Kyoto Encyclopedia of Genes and Genomes (KEGG) pathways ebriched in 56 DEERGs

To obtain the functions of these 56 DEERGs, GO function analysis of these 56 genes showed that they were involved in histone modification, chromatin organization and peptidyl-lysine modification and so on (Fig. 2A-B). KEGG pathway analysis showed that these DEERGs were associated with viral carcinogenesis, homologous recombination, cell cycle and fanconi anemia pathway (Fig. 2C). Figure 2D indicated that BRCA1 and BRCA2 were simultaneously involved in homologous recombination and fanconi anemia pathway, and CDK1 and CHEK1 were correlated with pathways of cell cycle and viral carcinogenesis.

### Establishment and validation of the prognostic model

**Fig. 3:**
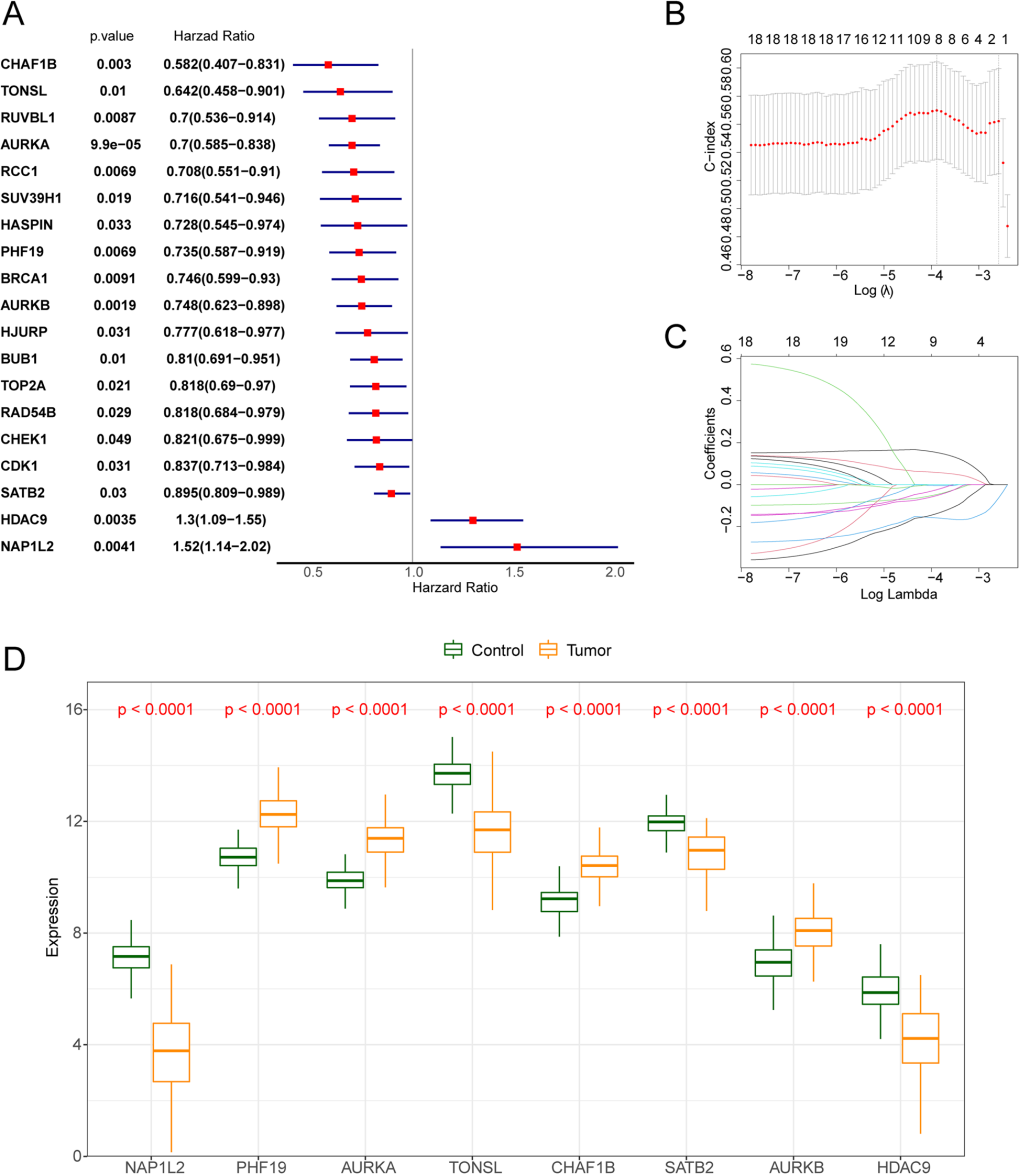
Identification of prognostic genes. **A** Univariate Cox regression analysis for 19 epigenetic-related genes (*p* < 0.05). **B-C** The plot of error plots for tenfold cross-validation (**B**) and gene coefficients (**C**) in least absolute shrinkage and selection operator (LASSO) analysis. **D** Boxplot of the expression level of 8 epigenetic-related genes in CRC groups and normal group

To construct epigenetic-related signature for survival prediction, we conducted univariate cox regression on the 56 DEERGs and selected 19 genes that were significantly linked with OS in training set (Fig. 3A). Inputting 19 genes into the LASSO model, eight genes were identified (Fig. 3B, C). Among them, PHF19, AURKA, CHAF1B and AURKB were up-regulated in the tumor group, NAP1L2, TONSL, SATB2 and HDAC9 were down-regulated in the tumor group (Fig. 3D). Furthermore, we determined the formula of risk score: (-0.047 × expression value of SATB2) + (0.058 × expression value of HDAC9) + (0.153 × expression value of NAP1L2) + (-0.024 × expression value of PHF19) + (-0.004 × expression value of AURKB) + (-0.052 × expression value of TONSL) + (-0.159 × expression value of AURKA) + (-0.138 × expression value of CHAF1B). Then CRC patients were classified as the high- and low-risk groups according to the median value of risk scores in the GSE40967.

**Fig. 4:**
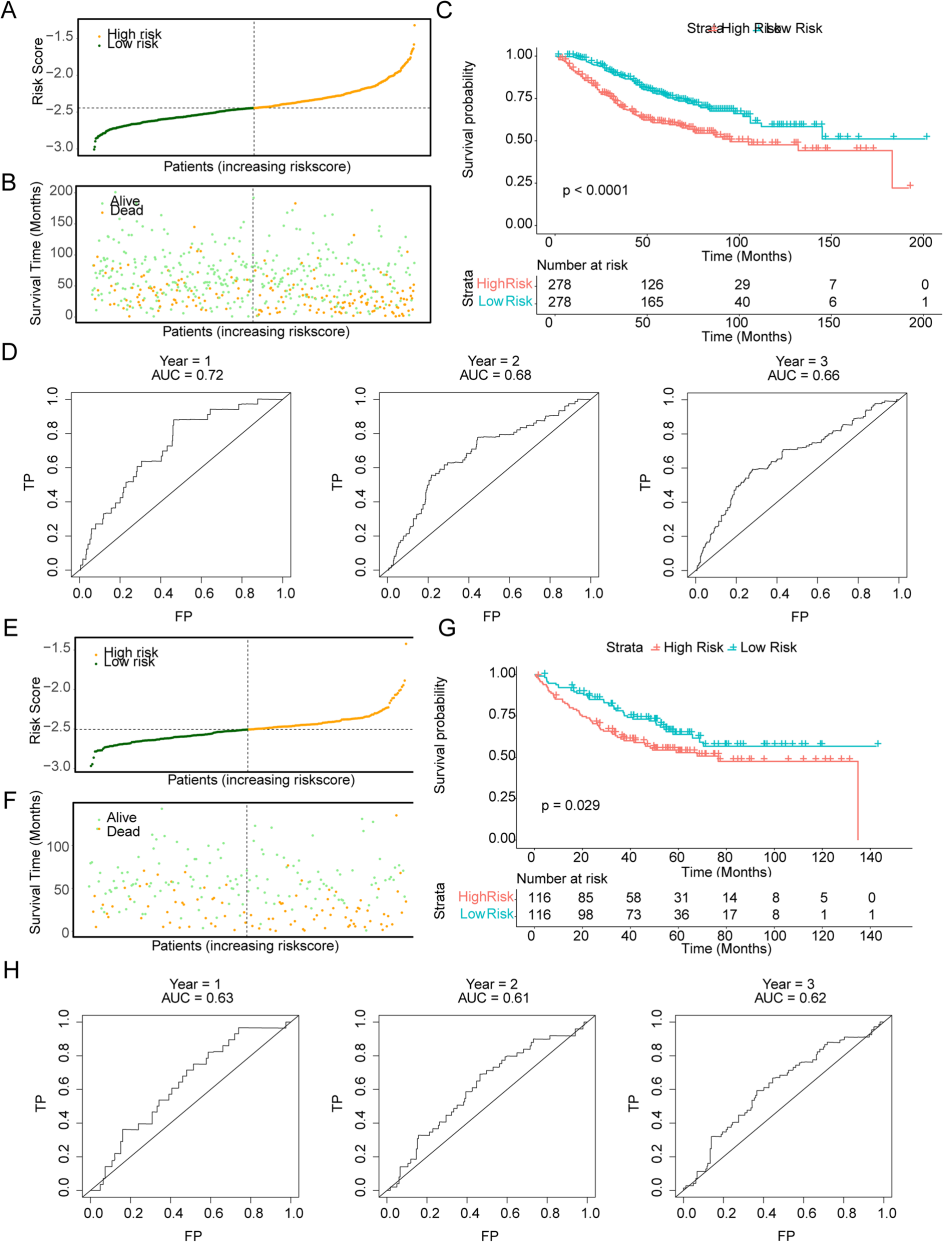
Construction and validation of epigenetic-related prognostic model. **A-B** Distribution of risk score, survival times and survival status in CRC patients. **C** Survival analysis between the high-risk and low-risk groups. **D** Receiver operating characteristic (ROC) curves of risk model for predicting survival in the GSE40967. AUC, area under the curve. **E–F** Distribution of risk score, survival times and survival status in the GSE17538. **G** Survival analysis between the high-risk and low-risk groups. **H** ROC curves of risk model for predicting survival in the GSE17538

Figure 4A, B demonstrated the risk scores and survival status between the high and low risk groups. Obviously, the high-risk group had poor prognosis of GC compared with low-risk group in the GSE40967 (Fig. 4C). ROC curve showed the AUC of risk score for 1-, 2-, 3- year survival status prediction was 0.72, 0.68, 0.66, indicated that risk score had moderate performance in predicting patient’s survival status (Fig. 4D). In the validation set, Kaplan–Meier analysis also showed a significant difference of overall survival (OS) (Fig. 4E-G) between two groups (high-risk and low-risk). AUC values of the risk model for 1–3 years in all the three cohorts were also greater than 0.6 (Fig. 4H).

### Clinical feature analysis and GSVA analysis

**Fig. 5:**
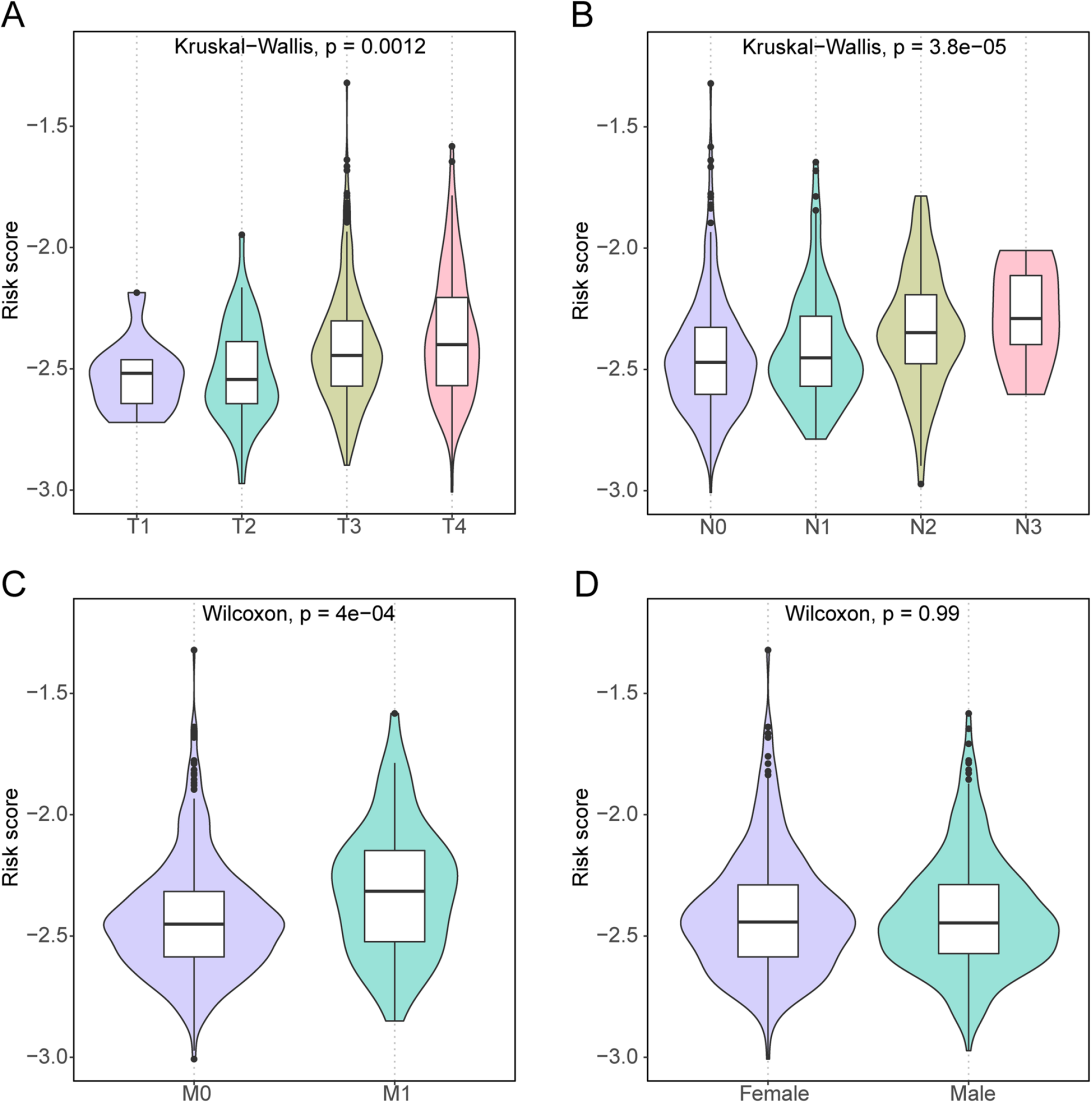
Differences in risk scores between subtypes of different clinical features. **A**: T stage; **B**: N stage; **C**: M stage; **D**: sex

We assessed the relevance between the clinicopathological traits and risk score, including gender and TNM stage. The risk score was significantly increased in advanced TNM stage cases (Fig. 5A-C) and the risk score was not significantly different in gender (Fig. 5D). The results showed that there was a powerful correlation between risk score and TNM stage.

**Fig. 6:**
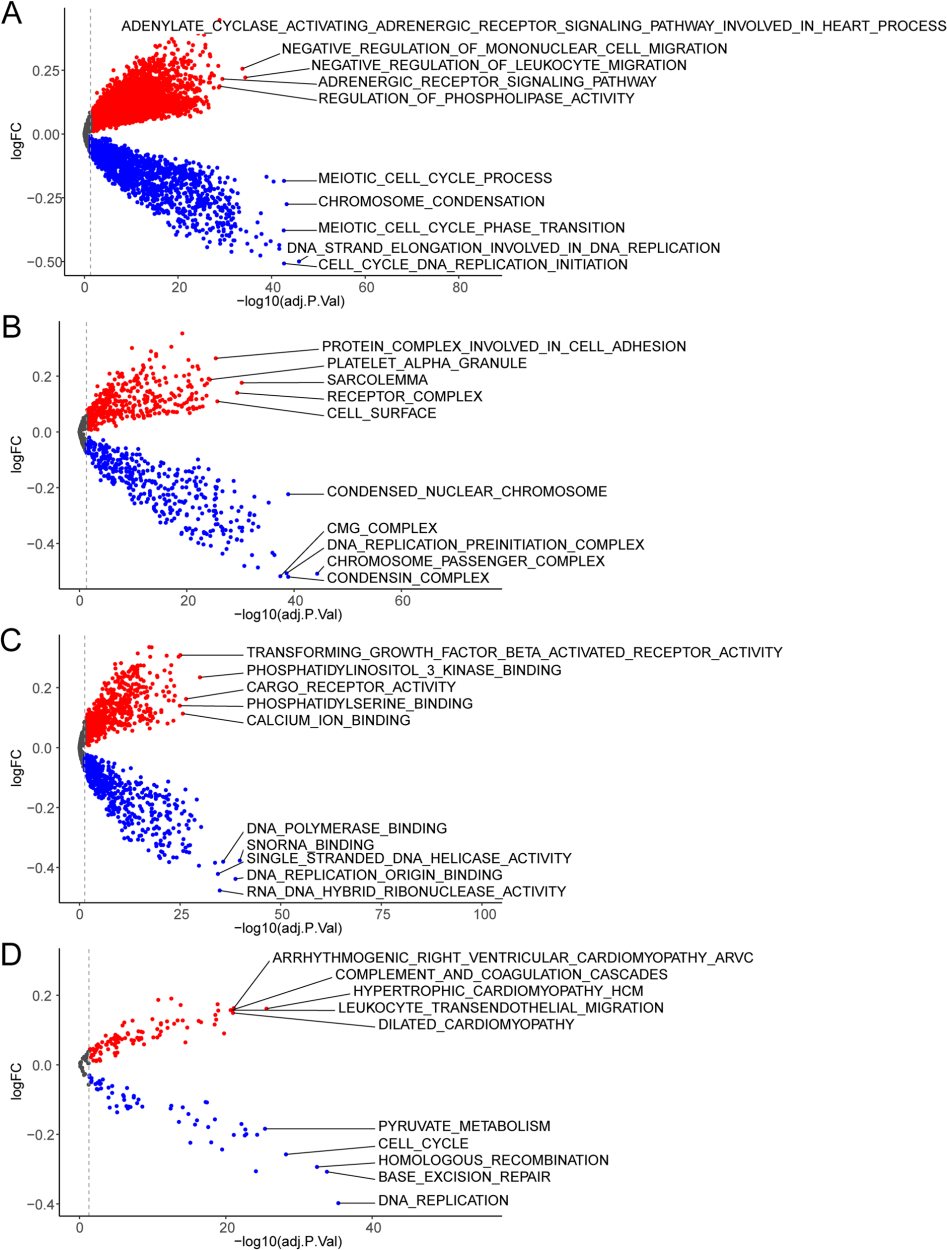
Genes function enrichment analysis. **A-D** GSVA between in the high-risk and low-risk group in CRC patients

We performed GSVA analysis with annotations of GO and KEGG gene sets to examine the potential biological functions between risk groups of CRC patients. The gene sets involved in hypertrophic cardiomyopathy HCM, negative regulation of leukocyte migration, sarcolemma and phosphatidylinositol 3 kinase binding were enriched in the high-risk group, while those related to DNA replication, DNA strand elongation involved in DNA replication, chromosome passenger complex and snoRNA binding were enriched in the low-risk group (Fig. 6A-D).

### Immune analysis of the high and low risk groups

**Fig. 7:**
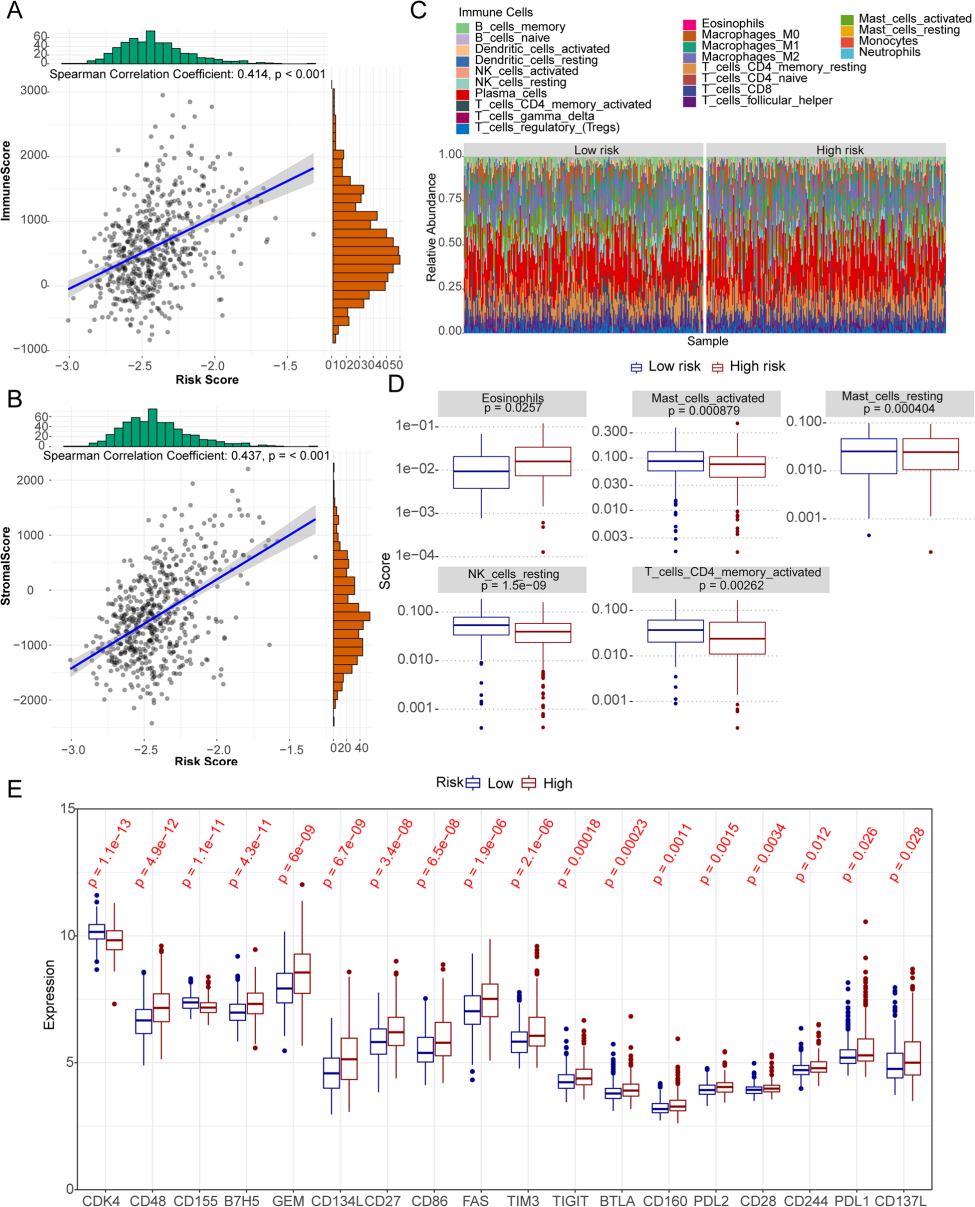
Immune infiltration analysis. **A**, **B** The relevance of risk score to immune score (**A**) and stromal score (**B**). **C** Differences in the proportions of immune cells between the high and low risk groups. **D** Boxplot of the difference of immune infiltration cells in in high and low risk groups. **E** Boxplot of the expression level of immune checkpoint in in high and low risk groups

We calculated immune/stromal scores and their correlation with risk scores. The results revealed that both the immunity score (cor = 0.414) and the stroma score (cor = 0.437) were significantly and positively correlated with the risk score (*p* < 0.05). (Fig. 7A, B).

Then we used CIBERSORT databases to assess the percentage of immune infiltrating cells in patients (Fig. 7C). Then we obtained 5 differential immune cells by CIBERSORT. The main differential immune cells between the risk groups (high and low) included NK cells resting, eosinophils, mast cells resting, T cells CD4 memory activated and mast cells active (Fig. 7D).

Furthermore, the expression of immune checkpoints were compared between the risk groups (high and low), the results showed that the expressions of CDK4, CD48, CD155, B7H5, GEM, CD134L, CD27, CD86, FAS, TIM3, TIGIT, BTLA, CD160, PDL2, CD28, CD244, PDL1 and CD137L were found to be significantly different between the two groups (Fig. 7E).

### Correlations of risk model genes with m6A and m5C associated genes

**Fig. 8:**
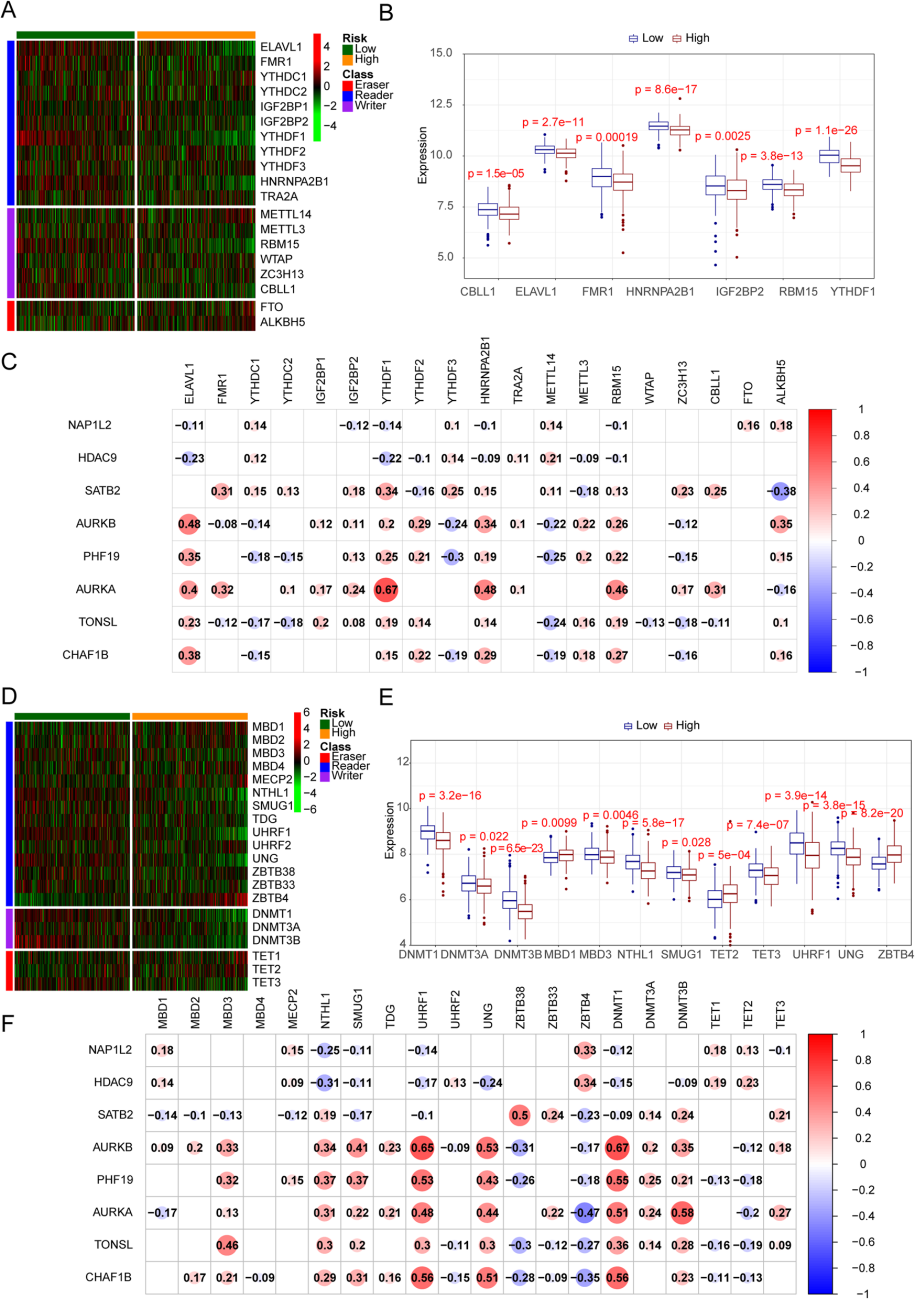
The relationship between risk model genes, m6A and m5C associated genes.** A** A heatmap showing the 19 m6A genes in high and low risk groups. **B-C** The relationship between m6A genes and 8 prognostic epigenetic-related genes. **D** A heatmap showing the 20 m5C genes in high and low risk groups. **E–F** The relationship between m5C genes and 8 prognostic epigenetic-related genes

We analyzed the expression patterns of 19 m6A regulators in CRC (Fig. 8A), and the results revealed that CBLL1, ELAVL1, FMR1, HNRNPA2B1, IGF2BP2, RBM15 AND YTHDF1 was significantly altered between the risk groups (high and low) (Fig. 8B). Then, correlation analysis was performed on the expression of 19 m6A-related genes and risk model genes (Fig. 8C), and we found AURKA had the most correlation to YTHDF1 (cor = 0.67). The correlation between other model genes and m6A-related genes were less than 0.5.

Then we evaluated the expressions of 20 m5C-related genes in CRC (Fig. 8D). The results revealed that MBD1, DNMT1, MBD3, SMUG1, ZBTB4, TET2, DNMT3A, TET3, UHRF1, DNMT3B, UNG and NTHL1 were significant difference between the risk groups (high and low) (Fig. 8E). We detected the correlation analysis between risk model genes and 20 m5C-related genes (Fig. 8F), and we found that AURKB was positively correlated with DNMT1(cor = 0.67), UHRF1 (cor = 0.65) and UNG (cor = 0.5). PHF19 was significantly positively correlated with DNMT1 (cor = 0.55) and UHRF1 (cor = 0.53), AURKA was significantly positively correlated with DNMT3B (cor = 0.58) and DNMT1 (cor = 0.51), CHAF18 was significantly positively correlated with DNMT1 (cor = 0.56), UHFR1 (cor = 0.56) and UNG (cor = 0.51) (Fig. 8F).

### Prediction of targeted drugs for AURKA, AURKB and HDAC9

**Fig. 9:**
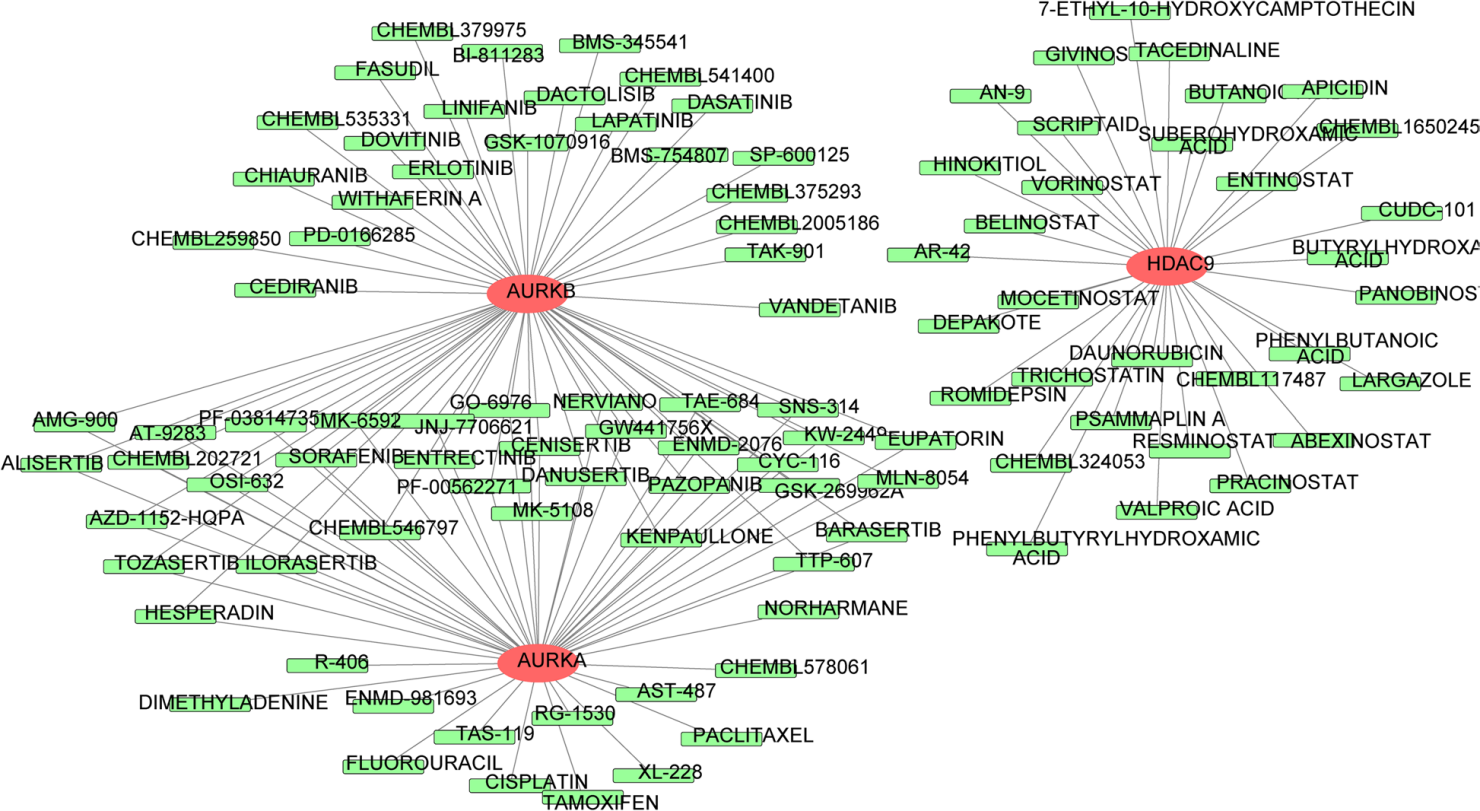
Prediction of drug sensitivity in the therapy of CRC. Red ovals represent genes and green rectangles represent targeted drugs

By means of eight model genes, we prediction of potential drugs for the treatment of CRC (Fig. 9). Only three genes, AURKA, AURKB and HDAC9, received the predicted drugs. A total of 137 drug-gene interaction pairs including 103 drugs and 3 model genes were found to have interactions. Among them, AURKA, AURKB and HDAC9 targeted by 47, 58, 32 drugs, respectively. Among them, pazopanib, danusertib, entrectinib and sorafenib targeted AURKA and AURKB. Givnostat, apicidin, belinostat and largazole targeted HDAC9.

### Analyses of independent prognostic and construction of the nomogram in CRC

**Fig. 10:**
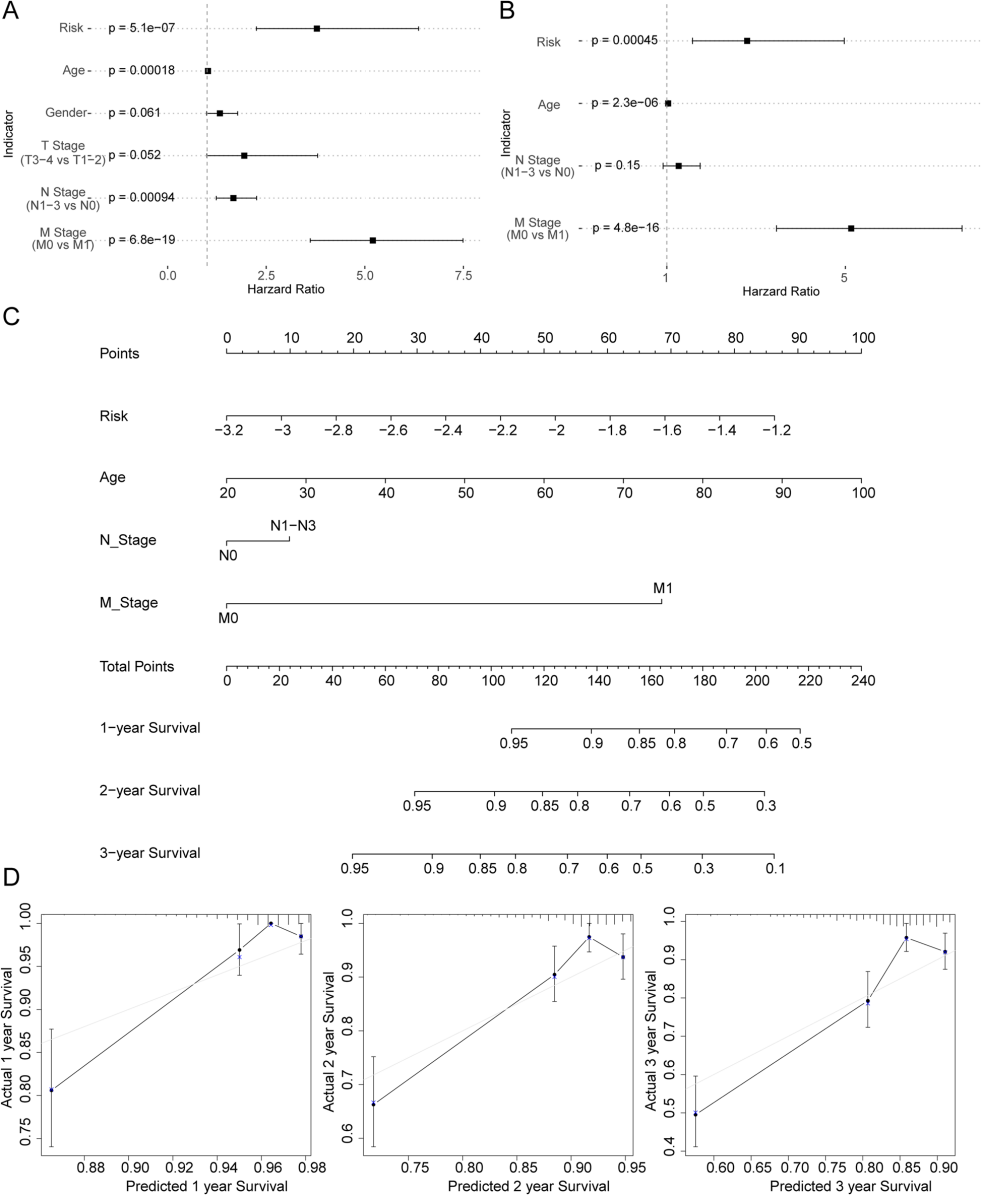
Independent prognostic analysis and construction of nomogram. **A** Univariate independent prognostic analysis in the training group. **B** Multivariate independent prognostic analysis in the training group. **C** A nomogram integrating clinical factors and risk score. **D** 1-,2-, and 3-year calibration plots of the nomogram

Importantly, TNM stage, age and risk score were significantly associated with prognosis in both univariate Cox analysis and mutivariate Cox analysis. Risk score, age, gender, TNM stage were included into univariate analysis (Fig. 10A), and risk score, age, T stage, N stage and M stage were used for multivariate analysis. The result indicated that risk score, age and N stage and M stage were independent prognostic factors in CRC (Fig. 10B). Thereafter, we constructed a nomogram to predict the 1-, 2-, and 3-year survival of CRC patients by using risk score, age N stage and M stage (Fig. 10C). The calibration curves for 1-, 2-, and 3-year (Fig. 10D) showed that the nomogram-predicted probability of survival was close to the actual survival.

### Experimental verification of model genes

**Fig. 11:**
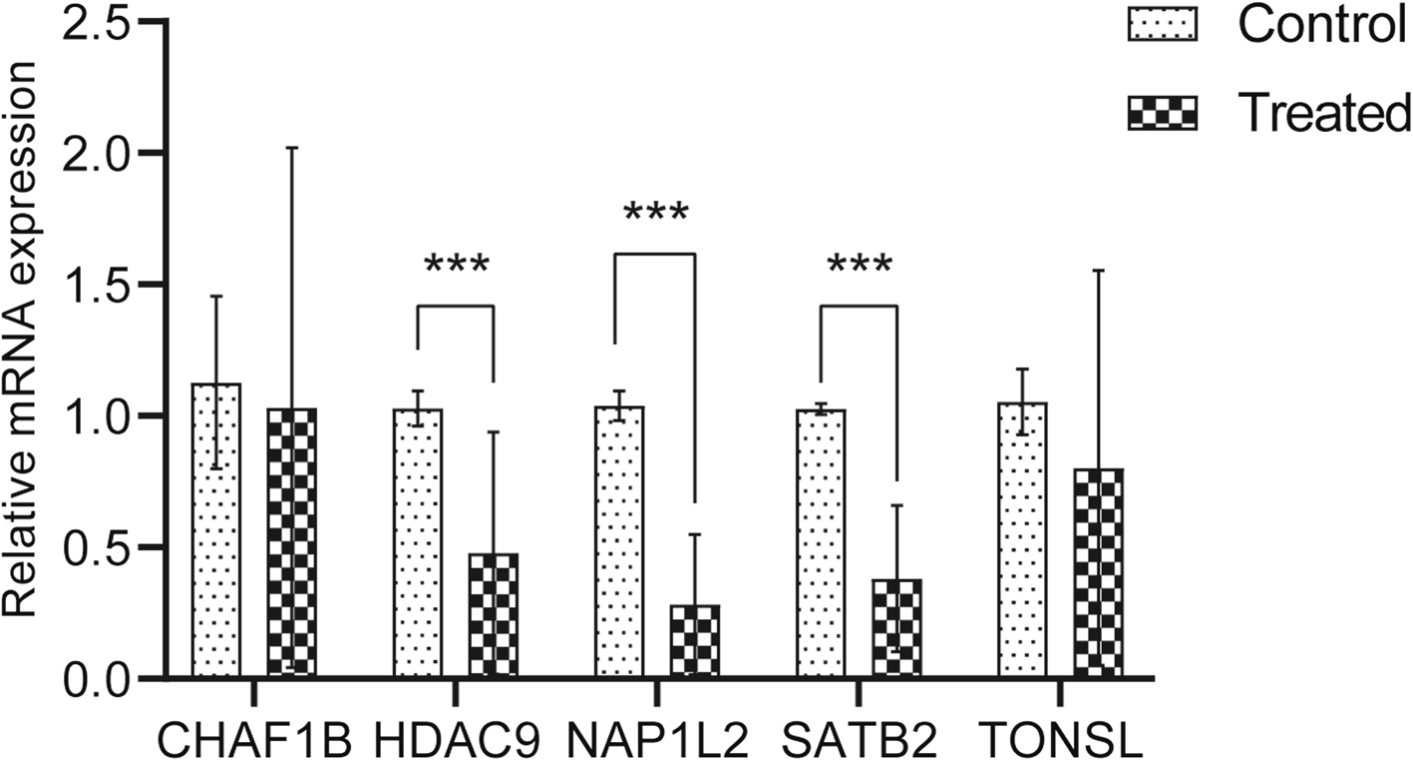
Verification of the expression of diagnosis-related genes. RT-qPCR assay for HDAC9, NAP1L2, SATB2, TONSL and CHAF1B in CRC (*n* = 20). *p < 0.05; ** *p* < 0.01; ***p < 0.001

The expressions of the 5 prognostic epigenetic-related genes were validated by quantitative real-time polymerase chain reaction (qRT-PCR) using 20 pairs of CRC and adjacent tissues. PCR experiments were conducted in which the expressions of HDAC9, NAP1L2 and SATB2 were significantly downregulated in CRC, but the differences between CHAF1B and TONSL in normal and disease samples are not obvious (Fig. 11, Supplementary Table 2).

## Discussion

Despite recent advancements in treatment, colorectal cancer still has a poor prognosis in advanced stages, indicating we must develop therapeutic targets in order to improve patient outcomes [34]. The identification of novel biomarkers and therapeutic targets is therefore crucial to improving the prognosis of colorectal cancer patients. Currently, no validated diagnostic and prognostic biomarkers for CRC have been identified. However, in the past, a number of epigenetic biomarkers could help predict and diagnose CRC, as well as provide prognosis [35]. But previous bioinformatics research only focused on single epigenetic-related genes but lacked extensive exploration, which had some limitations Many studies have revealed that epigenetic modification plays an important role in tumor progression. Undoubtedly, epigenetic mechanisms play a part in a wide range of cancers, and histone modification is one example of epigenetics that has drawn a lot of attention to scientists in recent years. Bioinformatics analysis showed that the above genes have effect in the prognosis of CRC, and the use of the obtained genes to construct risk models and predictive drugs for CRC patients provides clinical implications for targeted therapy.

During the analysis of this study, to ensure accuracy, we identified a total of 2906 differentially expressed DEGs between CRC and normal tissue samples. After overlapping DEGs with 720 ERGs that were obtained from EpiFactors database, we obtained 56 DEERGs. The KEGG pathways included viral carcinogenesis, homologous recombination, cell cycle and Fanconi anemia pathway. In addition, An analysis of GO functions revealed that these 56 genes played a role in histone modification, chromatin organization and peptidyl-lysine modification. The above pathways are closely associated with tumorigenesis, tumor metabolism, and metastasis and have been identified in CRC carcinogens based on KEGG and GO analysis [36, 37]. It is evident that epigenetics that affects gene activity and expression has been recognized as a critical role in the carcinogenesis [38].

Recently, *research* *on* histone modification, DNA methylation and chromatin organization and so on have become *increasingly* *popular* in tumor research [39]. It has been reported that dysfunction of histone modification plays a role in the etiology of a variety of human diseases, including gastrointestinal cancer, which involved in the activation of oncogenens and silence tumor suppressor genes [40–42]. Moreover, colorectal cancer is thought to develop as a consequence of altering histone modification patterns that lead to deregulation of gene expression [22, 43, 44]. Accordingly, many human diseases, including colon cancer, are linked to dysregulated phosphorylation, according to increasing numbers of studies [45]. As yet, it is rare for reports to discuss the association between histone phosphorylation and colorectal cancer. It has been indicated in several studies aberrant of phosphorylation histone as a factor in the pathogenesis of colorectal cancer [39]. For example, A study by Lee et al. found elevated H2AX phosphorylation in CRC tissues, which contributed to tumor behavior that was more aggressive, as well as poor CRC patient outcomes [46].

We examined eight prognostic epigenetic-related genes based on a risk model in this study, including NAP1L2, AURKB, TONSL, HDAC9, PHF19, CHAF1B, SATB2, AURKA. The analysis of showed that PHF19, AURKA, CHAF1B and AURKB were up-regulated in the tumor group, NAP1L2, TONSL, SATB2 and HDAC9 were down-regulated in the tumor group. As is known to all, Previously, four genes (AURKB, PHF19, SATB2, AURKA) were found to be associated with CRC [47–49]. However, there is no information on the role of NAP1L2, TONSL, HDAC9, and CHAF1B in colorectal cancer and were selected for further verification by qRT-PCR. Also, we selected certain genes such as SATB2 that is a promising biomarker for CRC. In the family of serine/threonine kinases, AURKA (Aurora kinase A) is a member. Korean colorectal adenocarcinoma patients may benefit from a AURKA level in order to predict poor outcomes [50]. Additionally, overexpression of AURKA in colorectal cancer liver metastases has been linked to poor outcomes [51]. AURKB has been proven to be correlated with supporting its potential role as a target in metastasis of CRC [52]. Many malignant tumors are affected by PHF19, which has a significant effect on prognosis [53]. Statistically, CRC patients with overexpression of PHF19 have a poorer survival rate [53]. It is evolutionarily conserved that the AT-rich sequence binding protein 2 (SATB2) plays a role in transcription. High SATB2 expression has been shown to predict good outcomes in colon cancer and modulate chemotherapy and radiation sensitivity [54]. By activating the pathway of NF‐κB that revealed a possible regulatory mechanism of NAP1L2 and impairing osteogenic potential through epigenetic regulation of histone acetylation at H3K14 [55]. Strikingly, 20 of the 21 significant SNPs resided in Histone Deacetylase 9 (HDAC9), an enzyme linked to epigenetic control of gene transcription and previously proposed to be an epigenetic switch for T-cell-mediated autoimmunity [56]. A key role played by SATB2 in integrating genetic and epigenetic signaling and the overexpression of PHF8 results in an upregulation the expression of SATB2 during osteogenic differentiation, we inferred that PHF8 might regulate SABT2 to activate osteogenic differentiation of BMSCs [57]. Using qRT-PCR, we confirmed that SATB2, HDAC9, NAP1L2 expression was down-regulated in the tumor group. Due to experimental conditions, sample size and tissue heterogeneity, the differences between CHAF1B and TONSL in normal and disease samples are not obvious, but we will continue to collect a large number of clinical samples to further verify our research results. Moreover, we analysis risk model genes between m5C-related genes and m6A- related genes. Obvious differences can be observed between 7 *m6A* and 12 m5C in the high- and low-risk groups. It was found that AURCK and YTHDF1 were positively correlated (*r* = 0.67), others were less than 0.5. In our results, the expression of AURKB and CHAF18 were both positively correlated with DNMT1, UHRF1 and UNG, and the expression of PHF19 was significantly positively correlated with DNMT1 and UHRF1, and the expression of AURKA was significantly positively correlated with DNMT3B and DNMT1. To achieve reliability, we also assessed the potential biological functions of the high-risk and low-risk groups using GSVA methods. Our results showed that hypertrophic cardiomyopathy HCM, negative regulation of leukocyte migration, sarcolemma and phosphatidylinositol 3 kinase binding were enriched in the high-risk group, and DNA replication, DNA strand elongation involved in DNA replication, chromosome passenger complex and snoRNA binding were enriched in the low-risk group and may be useful therapeutic targets. It is crucial for chromosome segregation and cytokinesis to be regulated by the chromosomal passenger complex (CPC), including Aurora B kinase, INCENP, Survivin and Borealin. Tuncel et al., study have shown that between Aurora B and Survivin expression has been verified to correlated with pathological features in colorectal carcinoma using immunohistochemistry [58]. Therefore, CRCs could benefit from diagnostic markers and therapeutic targets such as nuclear Aurora B and cytoplasmic Survivin. It has been suggested that CRC cells can grow unrestrained and become chemoresistance due to an overactivation of PI3K/AKT pathway. According to Lin et al. [59], Scutellaria barbata D. Don was able to inhibit CRC chemoresistance by suppressing the PI3K/AKT pathway. which could be a promising therapeutic target for CRC.

Additionally, the immune characteristics of all patients were discussed according to their risk scores and divided into low- and high-risk groups. The difference of immune cells in high and low risk groups mainly included eosinophils, mast cells active, mast cells resting, NK cells resting and T cells CD4 memory activated. It has been demonstrated that SETDB1 could activate the BATF3/PD-L1 axis by inhibiting FOSB-mediated miR-22 and promote immune evasion in CRC, which provides a better understanding of the mechanisms underlying immune evasion in CRC [60]. There was a significant changes in the proportions and functional states of T cells and B cells in tumor tissues when compared to those of paired non-tumor tissues [61]. It has been reported that there is an association between many immune cells and colorectal cancer prognosis [62]. It has been demonstrated in much more research that high immune cell infiltration is related to increased clinical symptoms and cure rates in CRC [63, 64]. Moreover, according to a new study, immune cell subtypes are associated with prognoses in CRC patients, giving the study potential clinical prognostic value [65]. Eosinophils, as the bone marrow-derived cells, reported that is related to antitumorigenic roles in CRC [66]. Previous studies have demonstrated that peritumoral eosinophils can serve as a prognostic indicator for CRC [67]. The CD4 + T cell plays an essential role in orchestrating antitumor immunity and promoting protective immunity [68]. Changes in M1 and M2 macrophages, resting and activated NK cells and activated mast cells all affect survival in CRC patients.

Based on bioinformatics analysis of this study is lack of the support from other experiment data, although we performed RT-qPCR assays, the lack of support from other experimental data are some of the limitations of our study. However, our study identified 8 prognostic epigenetic-related genes of CRC and developed a risk score model and a nomogram that can be used to predict prognosis.

## Conclusions

In this study, we constructed an epigenetic-related 8-gene signature by univariate and LASSO regression analysis. The Kaplan–Meier and Roc curve were used to analysis the accuracy of the model. Finally, the risk model is combined with the clinical characteristics of CRC patients to perform univariate and multivariate cox regression analysis to obtain independent risk factors and draw nomogram. To explore the potential value of epigenetics in therapeutic options and provide meaningful clinical implications for targeted therapy in CRC.

### Supplementary Information

**Additional file 1: Supplementary Table 1.** Gene and primer information.

** Additional file 2.** PRC model gene expression.

## Data Availability

The datasets used and/or analyzed during the current study can be made available from the corresponding author on reasonable request. We obtained the mRNA sequencing data of 203 CRC simples and 160 controls from Gene Expression Omnibus (GEO) database (https://www.ncbi.nlm.nih.gov/geo/). The relevant information involved in this study has been integrated into EpiFactors database (http://epifactors.autosome.ru) and DGIdb database (www.dgidb.org).

## Abbreviations

CRC: Colorectal cancer
ERGs: Epigenetic-related genes
DEGs: Differentially expressed genes
DEERGs: Differentially expressed epigenetic-related genes
OS: Overall survival
QRT-PCR: Quantitative real-time polymerase chain reaction

## References

[1] Torre LA, Bray F, Siegel RL, Ferlay J, Lortet-Tieulent J, Jemal A (2015). Global cancer statistics, 2012. CA CANCER J CLIN.

[2] Kuipers EJ, Grady WM, Lieberman D, Seufferlein T, Sung JJ, Boelens PG, van de Velde (2015). Colorectal cancer. Nat Rev Dis Primers.

[3] Zhang C, Zeng C, Xiong S, Zhao Z, Wu G (2022). A mitophagy-related gene signature associated with prognosis and immune microenvironment in colorectal cancer. Sci Rep.

[4] Elrebehy MA, Al-Saeed S, Gamal S, El-Sayed A, Ahmed AA, Waheed O (2022). miRNAs as cornerstones in colorectal cancer pathogenesis and resistance to therapy: a spotlight on signaling pathways interplay - a review. Int J Biol Macromol.

[5] Meneses-Morales I, Izquierdo-Torres E, Flores-Peredo L, Rodríguez G, Hernández-Oliveras A, Zarain-Herzberg Á (2019). Epigenetic regulation of the human ATP2A3 gene promoter in gastric and colon cancer cell lines. Mol Carcinog.

[6] Xing XL, Yao ZY, Xing C (2021). Gene expression and DNA methylation analyses suggest that two immune related genes are prognostic factors of colorectal cancer. BMC Med Genomics.

[7] NazemalhosseiniMojarad E, Kuppen PJ, Aghdaei HA, Zali MR (2013). The CpG island methylator phenotype (CIMP) in colorectal cancer. Gastroenterol Hepatol Bed Bench.

[8] Lu Y, Chan YT, Tan HY, Li S, Wang N, Feng Y (2020). Epigenetic regulation in human cancer: the potential role of epi-drug in cancer therapy. Mol Cancer..

[9] Alzrigat M, Párraga AA, Jernberg-Wiklund H (2018). Epigenetics in multiple myeloma: from mechanisms to therapy. Semin Cancer Biol.

[10] Abdelaziz N, Therachiyil L, Sadida HQ (2023). Epigenetic inhibitors and their role in cancer therapy. Int Rev Cel Mol Bio..

[11] Saleh R, Toor SM, Sasidharan Nair V (2020). Role of Epigenetic modifications in inhibitory immune checkpoints in cancer development and progression. Front Immunol.

[12] Lazennec G, Lam PY (2016). Recent discoveries concerning the tumor - mesenchymal stem cell interactions. Biochim Biophys Acta.

[13] El Bairi K, Tariq K, Himri I, Jaafari A, Smaili W, Kandhro AH, Gouri A, Ghazi B (2018). Decoding colorectal cancer epigenomics. Cancer Genetics..

[14] Farkas SA, Vymetalkova V, Vodickova L, Vodicka P, Nilsson TK (2014). DNA methylation changes in genes frequently mutated in sporadic colorectal cancer and in the DNA repair and Wnt/β-catenin signaling pathway genes. Epigenomics.

[15] Vymetalkova V, Vodicka P, Pardini B, Rosa F, Levy M, Schneiderova M (2016). Epigenome-wide analysis of DNA methylation reveals a rectal cancer-specific epigenomic signature. Epigenomics.

[16] Nguyen HT, Duong HQ (2018). The molecular characteristics of colorectal cancer: Implications for diagnosis and therapy. Oncol Lett.

[17] Hong SN (2018). Genetic and epigenetic alterations of colorectal cancer. Intest Res.

[18] Zhang D, Guo S, Schrodi SJ (2021). Mechanisms of DNA methylation in virus-host interaction in hepatitis B infection: pathogenesis and oncogenetic properties. Int J Mol Sci..

[19] Leone V, Ali A, Weber A, Tschaharganeh DF, Heikenwalder M (2021). Liver inflammation and hepatobiliary cancers. Trends Cancer..

[20] Chen Y, Ren B, Yang J, Wang H, Yang G, Xu R (2020). The role of histone methylation in the development of digestive cancers: a potential direction for cancer management. Signal Transduct Target Ther.

[21] Qin J, Wen B, Liang Y (2019). Histone modifications and their role in colorectal cancer (review). Pathol Oncol Res.

[22] Karczmarski J, Rubel T, Paziewska A, Mikula M, Bujko M, Kober P (2014). Histone H3 lysine 27 acetylation is altered in colon cancer. Clin Proteomics.

[23] Gebrekiristos M, Melson J, Jiang A, Buckingham L (2022). DNA methylation and miRNA expression in colon adenomas compared with matched normal colon mucosa and carcinomas. Int J Exp Pathol.

[24] Vogelstein B, Fearon ER, Hamilton SR, Kern SE, Preisinger AC, Leppert M (1988). Genetic alterations during colorectal-tumor development. N Engl J Med.

[25] Siskova A, Cervena K, Kral J, Hucl T, Vodicka P, Vymetalkova V (2022). Colorectal adenomas-genetics and searching for new molecular screening biomarkers. Int J Mol Sci..

[26] Kalmár A, Péterfia B, Hollósi P, Galamb O, Spisák S, Wichmann B (2015). DNA hypermethylation and decreased mRNA expression of MAL, PRIMA1, PTGDR and SFRP1 in colorectal adenoma and cancer. BMC Cancer.

[27] Medvedeva YA, Lennartsson A, Ehsani R, Kulakovskiy IV, Vorontsov IE, Panahandeh P (2015). EpiFactors: a comprehensive database of human epigenetic factors and complexes. Database (Oxford)..

[28] Love MI, Huber W, Anders S (2014). Moderated estimation of fold change and dispersion for RNA-seq data with DESeq2. Genome Biol.

[29] The Gene Ontology Consortium (2017). Expansion of the gene ontology knowledgebase and resources. Nucleic Acids Res.

[30] Kanehisa M, Goto S (2000). KEGG: kyoto encyclopedia of genes and genomes. Nucleic Acids Res.

[31] Yoshihara K, Shahmoradgoli M, Martínez E, Vegesna R, Kim H, Torres-Garcia W (2013). Inferring tumour purity and stromal and immune cell admixture from expression data. Nat Commun.

[32] Cotto KC, Wagner AH, Feng YY, Kiwala S, Coffman AC, Spies G (2018). DGIdb 3.0: a redesign and expansion of the drug-gene interaction database. Nucleic Acids Res.

[33] Schmittgen TD, Livak KJ (2008). Analyzing real-time PCR data by the comparative C(T) method. Nat Protoc.

[34] Zafari N, Bathaei P, Velayati M (2023). Integrated analysis of multi-omics data for the discovery of biomarkers and therapeutic targets for colorectal cancer. Comput Biol Med.

[35] Ray SK, Mukherjee S (2023). Assimilating epigenetics and transcriptomics for the identification of prognostic novel biomarkers and imminent targets in colorectal carcinoma with therapeutic potential. Curr Mol Med.

[36] Chen L, Zhang YH, Lu G, Huang T, Cai YD (2017). Analysis of cancer-related lncRNAs using gene ontology and KEGG pathways. Artif Intell Med.

[37] Chasov V, Zaripov M, Mirgayazova R, Khadiullina R, Zmievskaya E, Ganeeva I (2021). Promising new tools for targeting P53 mutant cancers: humoral and cell-based immunotherapies. Front Immunol.

[38] Huo M, Zhang J, Huang W (2021). Interplay among metabolism, epigenetic modifications, and gene expression in cancer. Front Cell Dev Biol..

[39] Yang Z, Huang D, Meng M (2022). BAF53A drives colorectal cancer development by regulating DUSP5-mediated ERK phosphorylation. Cell Death Dis.

[40] AlaskharAlhamwe B, Khalaila R, Wolf J, von Bülow V, Harb H, Alhamdan F (2018). Histone modifications and their role in epigenetics of atopy and allergic diseases. Allergy Asthma Clin Immunol.

[41] He H, Hu Z, Xiao H, Zhou F, Yang B (2018). The tale of histone modifications and its role in multiple sclerosis. Hum Genomics.

[42] Biswas S, Rao CM (2017). Epigenetics in cancer: fundamentals and beyond. Pharmacol Ther.

[43] Bardhan K, Paschall AV, Yang D, Chen MR, Simon PS, Bhutia YD (2015). IFN induces DNA methylation-silenced GPR109A expression via pSTAT1/p300 and H3K18 acetylation in colon cancer. Cancer Immunol Res.

[44] Yu D, Li Z, Gan M, Zhang H, Yin X, Tang S (2015). Decreased expression of dual specificity phosphatase 22 in colorectal cancer and its potential prognostic relevance for stage IV CRC patients. Tumor Biol.

[45] Cordeiro MH, Smith RJ, Saurin AT (2018). A fine balancing act: A delicate kinase-phosphatase equilibrium that protects against chromosomal instability and cancer. Int J Biochem Cell Biol.

[46] Lee YC, Yin TC, Chen YT, Chai CY, Wang JY, Liu MC (2015). High expression of phospho-H2AX predicts a poor prognosis in colorectal cancer. Anticancer Res.

[47] Kasap E, Gerceker E, Boyacıoglu SÖ, Yuceyar H, Yıldırm H, Ayhan S (2016). The potential role of the NEK6, AURKA, AURKB, and PAK1 genes in adenomatous colorectal polyps and colorectal adenocarcinoma. Tumour Biol.

[48] Li QL, Lin X, Yu YL (2021). Genome-wide profiling in colorectal cancer identifies PHF19 and TBC1D16 as oncogenic super enhancers. Nat Commun.

[49] Cígerová V, Adamkov M, Drahošová S, Grendár M (2021). Immunohistochemical expression and significance of SATB2 protein in colorectal cancer. Ann Diagn Pathol.

[50] Koh HM, Jang BG, Hyun CL, Kim YS, Hyun JW, Chang WY (2017). Aurora kinase A is a prognostic marker in colorectal adenocarcinoma. J Pathol Transl Med.

[51] Goos JA, Coupe VM, Diosdado B, Delis-Van Diemen PM, Karga C, Beliën JA (2013). Aurora kinase A (AURKA) expression in colorectal cancer liver metastasis is associated with poor prognosis. Brit J Cancer..

[52] Pohl A, Azuma M, Zhang W, Yang D, Ning Y, Winder T (2011). Pharmacogenetic profiling of Aurora kinase B is associated with overall survival in metastatic colorectal cancer. Pharmacogenomics J.

[53] Li P, Sun J, Ruan Y (2021). High PHD Finger Protein 19 (PHF19) expression predicts poor prognosis in colorectal cancer: a retrospective study. Peer J.

[54] Eberhard J, Gaber A, Wangefjord S, Nodin B, Uhlén M, Ericson Lindquist K (2012). A cohort study of the prognostic and treatment predictive value of SATB2 expression in colorectal cancer. Brit J Cancer.

[55] Hu M, Xing L, Zhang L (2022). NAP1L2 drives mesenchymal stem cell senescence and suppresses osteogenic differentiation. Aging Cell.

[56] Barry JD, Fagny M, Paulson JN (2018). Histopathological Image QTL discovery of immune infiltration variants. iScience..

[57] Han Q, Yang P, Wu Y (2015). Epigenetically modified bone marrow stromal cells in silk scaffolds promote craniofacial bone repair and wound healing. Tissue Eng PT A.

[58] Tuncel H, Shimamoto F, Kaneko Guangying Qi H, Aoki E, Jikihara H, Nakai S (2012). Nuclear Aurora B and cytoplasmic Survivin expression is involved in lymph node metastasis of colorectal cancer. Oncol Lett..

[59] Lin J, Feng J, Yang H, Lin J, Feng J, Yang H (2017). Scutellaria barbata D. Don inhibits 5-fluorouracil resistance in colorectal cancer by regulating PI3K/AKT pathway. Oncol Rep..

[60] Tian J, Wang W, Zhu J (2022). Histone Methyltransferase SETDB1 promotes immune evasion in colorectal cancer via FOSB-mediated downregulation of MicroRNA-22 through BATF3/PD-L1 pathway. J Immunol Res..

[61] Wang W, Zhong Y, Zhuang Z (2021). Multiregion single-cell sequencing reveals the transcriptional landscape of the immune microenvironment of colorectal cancer. Clin Transl Med.

[62] Malka D, Lièvre A, André T, Taïeb J, Ducreux M, Bibeau F (2020). Immune scores in colorectal cancer: Where are we?. Eur J Cancer.

[63] Deng S, Zhu Q, Chen H (2023). Screening of prognosis-related Immune cells and prognostic predictors in colorectal cancer patients. BMC Cancer.

[64] Adams S, Gray RJ, Demaria S, Goldstein L, Perez EA, Shulman LN (2014). Prognostic value of tumor-infiltrating lymphocytes in triple-negative breast cancers from two phase III randomized adjuvant breast cancer trials: ECOG 2197 and ECOG 1199. J Clin Oncol.

[65] Ding TT, Zeng CX, Hu LN, Yu MH (2022). Establishment of a prediction model for colorectal cancer immune cell infiltration based on the cancer genome atlas (TCGA) database. Beijing Da Xue Xue Bao Yi Xue Ban.

[66] Reichman H, Itan M, Rozenberg P, Yarmolovski T, Brazowski E, Varol C (2019). Activated eosinophils exert antitumorigenic activities in colorectal cancer. Cancer Immunol Res.

[67] Ramadan S, Saka B, Yarikkaya E, Bilici A, Oncel M (2020). The potential prognostic role of peritumoral eosinophils within whole tumor-associated inflammatory cells and stromal histological characteristics in colorectal cancer. Pol J Pathol.

[68] Ben Khelil M, Godet Y, Abdeljaoued S, Borg C, Adotévi O, Loyon R (2022). Harnessing antitumor CD4^+^ T cells for cancer immunotherapy. Cancers (Basel).

