# Supplementary material for: Development and validation of epigenetic modification-related signals for the diagnosis and prognosis of colorectal cancer
Source: BMC Genomics. 2024 Jan 11;25:51. doi: 10.1186/s12864-023-09815-2 (PMC10782594; doi:10.1186/s12864-023-09815-2)
Supplement: Supplementary file 1 — Additional file 1: Supplementary Table 1. Gene and primer information. [file 12864_2023_9815_MOESM1_ESM.docx]

Supplementary Table 1 Gene and primer information

| Gene | Accession number | Primer sequence | Product length | Annealing  temperature |
| --- | --- | --- | --- | --- |
| *NAP1L2* | NM_021963.4 | F: GTTCTCAAAGCCTCAGCACCA  R: CAAAGGACCGTACACGCCTAA | 179 | 60 |
| *HDAC9* | NM_001204144.3 | F: CTTGTAGCTGGTGGAGTTCCC  R: CTCTGTCTTCCTGCATCGCCT | 328 | 60 |
| *SATB2* | NM_001172509.2 | F: GGAGGAGTCAAGGCATCACC  R: GCCTTCCTCGCTGTCGTTCT | 448 | 60 |
| *TONSL* | NM_013432.5 | F: GCAGAGCAATGACGAGGTGTT  R: TGCGGTAGCGGTCAGTCAA | 71 | 60 |
| *CHAF1B* | NM_005441.3 | F: GATGAGTCTGCCCTACCGC  R: AACTTGGTGGAGTGTCCGTCTT | 608 | 60 |
| *Actin* | NM_001101.3 | F:TGGCACCCAGCACAATGAA  R:AGGGTGTAACGCAACTAAGTCATAG | 200 | 60 |
